# Cutis Marmorata Telangiectatica Congenita Associated with Hemiatrophy

**DOI:** 10.1155/2020/8813809

**Published:** 2020-10-07

**Authors:** Alexander K. C. Leung, Joseph M. Lam, Kin Fon Leong

**Affiliations:** ^1^The University of Calgary and Pediatric Consultant at The Alberta Children's Hospital, Calgary, Alberta, T2M 0H5, Canada; ^2^Department of Dermatology and Skin Sciences, University of British Columbia and Consultant Pediatric Dermatologist at the BC Children's Hospital Vancouver, Vancouver, British Columbia, V6H 3V4, Canada; ^3^Pediatric Institute, Kuala Lumpur General Hospital, Kuala Lumpur, Malaysia

## Abstract

Cutis marmorata telangiectatica congenita is characterized by the presence of a bluish-purple reticulated cutaneous vascular network on the skin intermixed with telangiectasia and occasionally prominent veins at birth. Areas of the skin within the reticulated cutaneous vascular network may be normal, erythematous, atrophic, and, at times, ulcerated. Areas of ulcerations and focal cutaneous and subcutaneous atrophy occasionally occur resulting in body asymmetry. On the other hand, cutaneous and subcutaneous atrophy, extensive and severe enough leading to hemiatrophy, of the entire limb is rare. A search of the English literature revealed only eight documented cases to which we are adding two more cases.

## 1. Introduction

Hemiatrophy refers to wasting or loss of tissue on one side of the body. The condition may result from congenital hemiatrophy due to intrauterine insult, contralateral cerebral insult, amyotrophic lateral sclerosis, syringobulbia, ipsilateral peripheral neuropathy, trauma to the muscles on one side of the body, prolonged immobilization on one side of the body (disuse atrophy), congenital or acquired lipodystrophy, unilateral morphea, Duchenne muscular dystrophy, facioscapulohumeral muscular dystrophy, or cutis marmorata telangiectatica congenita [1–4]. Hemiatrophy may also be a manifestation of certain syndromes such as Parry–Romberg syndrome, Dyke–Davidoff–Masson syndrome, and hemidystonia-hemiatrophy syndrome [5]. Documented cutis marmorata telangiectatica congenita associated with hemiatrophy is rare. A PubMed search of the English literature using the key terms “cutis marmorata telangiectatica congenita” AND “hemiatrophy” revealed only eight cases, to which we are adding two cases to alert readers of such association.

## 2. Case Reports

### 2.1. Case 1

A 2-week-old Indian girl presented with fishnet dark red to purplish atrophic patches over her left lower extremity, which had been present since birth. The skin lesion did not fade with warming. Her parents had also noticed a discrepancy in the size of her lower limbs, with relative atrophy of the left lower limb. The infant was born to a gravida 2, para 1, 30-year-old mother at 38 weeks of gestation. The pregnancy was uncomplicated, and the mother was not on any medications. Cesarean section was performed because of fetal distress. The Apgar score was 6 at 1 minute and 9 at 5 minutes. Her birth weight was 2.7 kg, length was 49.5 cm, and head circumference was 34.6 cm. There was no family history of vascular malformations.

**Figure 1:**
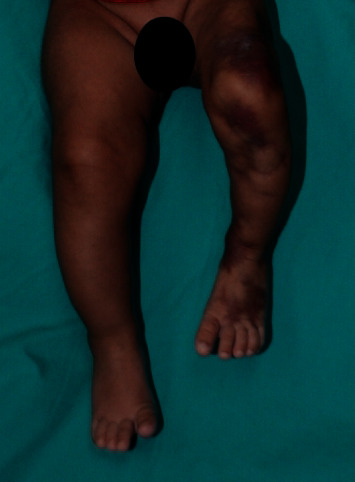
Cutis marmorata telangiectatica congenita on the left lower limb associated with ipsilateral hemiatrophy (anterior view).

**Figure 2:**
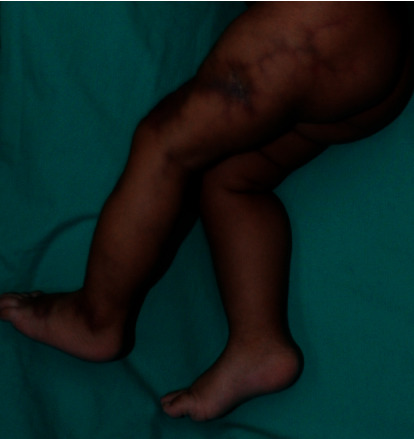
Cutis marmorata telangiectatica congenita on the left lower limb associated with ipsilateral hemiatrophy (lateral view).

On examination, the infant appeared well and was in no apparent distress. Her weight was 3.02 kg, length was 49.8 cm, and head circumference was 49.9 cm. Dermatologic examination revealed multiple reticulated dark red to purplish atrophic plaques extending from the left foot to the groin (Figures 1 and 2). Cutaneous atrophy was also noted. The length of her lower limbs, measured from the anterior superior iliac spine to the medial malleolus, was 19.9 cm on the left side and 22.4 cm on the right side. The midthigh circumference was 21.2 cm on the left and 24.5 cm on the right. The midcalf circumference was 14.2 cm on the left and 16.8 cm on the right. The rest of the examination, including the neurologic examination, was normal.

A cranial ultrasound was performed and was found to be normal. A diagnosis of cutis marmorata telangiectatica congenita on the left lower limb associated with ipsilateral hemiatrophy was made.

### 2.2. Case 2

A 1-week-old Chinese girl presented with marbled dark red atrophic plaques over her right lower extremity, which had been fully established since birth. The lesion did not fade with warming. The right lower limb was noted to be smaller than the left lower limb. She was born to a 24-year-old primiparous mother at 38 weeks gestation following an uncomplicated pregnancy and normal vaginal delivery. The Apgar score was 5 at 1 minute and 9 at 5 minutes. Her birth weight was 3.5 kg, length was 51.2 cm, and head circumference was 35 cm. Family medical history was noncontributory.

**Figure 3:**
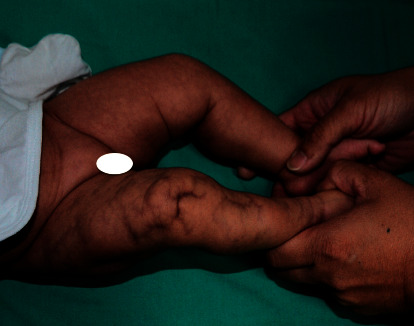
Cutis marmorata telangiectatica congenita on the right lower limb associated with ipsilateral hemiatrophy.

Physical examination revealed multiple marbled dark red atrophic plaques on right lower extremity (Figure 3). The length of her lower limbs, measured from the anterior superior iliac spine to the medial malleolus, was 21.6 cm on the left side and 21.4 cm on the right side. The midthigh circumference was 25.2 cm on the left and 24.1 cm on the right. The midcalf circumference was 17.5 cm on the left and 16.8 cm on the right. The rest of the examination was normal.

Cranial ultrasound was performed and was found to be normal. A diagnosis of cutis marmorata telangiectatica congenita on the right lower limb associated with ipsilateral hemiatrophy was made.

## 3. Discussion

Cutis marmorata telangiectatica congenita was first recognized as a distinct vascular defect in 1922 by Dutch pediatrician Van Lohuizen who described a child with skin lesions resembling livedo reticularis that were accompanied by telangiectases and superficial ulcerations [6]. Cutis marmorata telangiectatica congenita is characterized by the presence of a reticulated cutaneous vascular network of bluish-purple color on the skin intermixed with telangiectasia and occasionally prominent veins at birth [7, 8]. Areas of the skin within the reticulated cutaneous vascular network may be normal, erythematous, atrophic, and, at times, ulcerated [9]. Crying and exposure to cold environments usually intensify the vascular pattern, but the mottling does not resolve when the skin is warmed. Compared to cutis marmorata, the discoloration in cutis marmorata telangiectatica congenita is bluish-purple rather than red and the lesion is more intense and is persistent [8]. Cutis marmorata telangiectatica congenita can be generalized over the entire body or localized to a specific area of the body. When localized, the lesion typically affects one extremity, although the trunk and face may also be involved. Characteristically, the lesion does not cross the midline [8].

In one study of 35 children with cutis marmorata telangiectatica congenita, associated anomalies, usually minor and sometimes questionable, were noted in 28 (80%) children [10]. Areas of ulcerations and focal cutaneous and subcutaneous atrophy resulting in body asymmetry are well known [8, 11–13]. On the other hand, cutaneous and subcutaneous atrophy, extensive and severe enough to result in hemiatrophy, of the entire limb is rare. A search of the English literature revealed only eight documented cases, to which we are adding two more cases (Table 1). In 1970, Fitzsimmons and Starks reported a female neonate who presented at birth with bilateral cutis marmorata telangiectatica congenita on the arms, legs, and trunk, which was more marked on the left side [14]. She was noted to have hemiatrophy of the left side of the body at four months of age. There was a 2.5 cm difference in the circumference of the left arm and the left leg compared with the contralateral limbs. The left side of the trunk, the left pectoral area, and the left shoulder area were also smaller than those on the right side. At 3 years and 11 months of age, measurements showed that the circumference of the upper part of the right arm was 1 cm less than that of the left, a right forearm circumference 0.7 cm less than that of the left, and a right thigh and calf circumference 0.3 cm less than that of the left. In 1971, Keipert and Nurse described a newborn male with cutis marmorata telangiectatica congenita on both legs (more marked on the right leg), right arm, and right midtrunk [15]. At two months of age, wasting of the right arm was noted. In addition, the right calf was smaller than the left calf. South and Jacobs reported a newborn girl with cutis marmorata telangiectatica congenita on her legs, feet, back, and chest [16]. The lesion on the chest was resolved in the neonatal period. The child also had hemiatrophy affecting the right side of the body. The authors, however, did not specify the age when the atrophy was noted or the specific site of the hemiatrophy. In 1984, Spraker et al. reported an infant girl whose mother noted atrophy of the entire left side of the body together with cutis marmorata telangiectatica congenita on the left face and the trunk when the infant was 2 weeks old [17]. In addition, the infant also had congenital generalized fibromatosis and porencephaly. In 2001, Avci reported a 7-year-old girl who was noted to have cutis marmorata telangiectatica congenita on most of her body at birth [18]. Soon, the family noted that her right leg was shorter and thinner than the left leg. Physical examination showed that the child's right lower extremity was 4.5 cm shorter than the left side. The right thigh circumference, measured 10 cm proximal to the superior pole of the patella, was 5.5 cm thinner than that on the left thigh. In 2011, Levy and Lam reported a three-month-old girl presented with cutis marmorata telangiectatica congenita on her right thigh at birth [19]. The girl was noted to have relative atrophy of the right thigh. The right thigh was smaller in girth than the left thigh. The length of her lower limbs, however, did not differ. In 2014, de Maio et al. reported a newborn infant with lesions of cutis marmorata telangiectatica congenita involving the whole body at birth [20]. The infant was subsequently noted to have left hemiatrophy affecting the left lower limb. The circumference of the right thigh (22.5 cm versus 21 cm) and the right leg (17 cm versus 15 cm) was greater than their counterparts. In 2018, Amaral et al. reported a 39-week-old male infant who presented at birth with cutis marmorata telangiectatica congenita in a typical right-sided segmental distribution [21]. At 6 months of age, the right lower limb was 1.5 cm shorter than the left lower limb and had a smaller diameter. There was a 3 cm and a 2.5 cm difference in the thigh and the leg diameter, respectively.

In most cases, clinical improvement of the cutaneous lesions occurs within the first two years of life, [22] although the lesions occasionally persist [22]. The associated hemiatrophy, on the other hand, tends to persist. The present cases suggest that hemiatrophy associated with cutis marmorata telangiectatica congenita is more common than being presently appreciated. Physicians should bear this in mind when encountering a child with cutis marmorata telangiectatica congenita.

## 4. Conclusion

Cutis marmorata telangiectatica congenita typically presents at birth with a persistent, reticulated, bluish-purple vascular network that may be localized to a specific area or generalized over the entire body. While the skin lesions tend to improve with time, the associated hemiatrophy tends to persist. The present cases show that hemiatrophy resulting from cutis marmorata telangiectatica congenita is more common than being presently appreciated. Physicians should bear this in mind when encountering a child with cutis marmorata telangiectatica congenita.

## Figures and Tables

**Table 1:** Cutis marmorata telangiectasis congenita with hemiatrophy.

Authors (year of publication)	Race/ethnicity	Sex	Age at presentation of CMTC	Site of CMTC	Age at presentation of hemiatrophy	Site of hemiatrophy
Fitzsimmons and Starks (1970) [14]	NS	F	Birth	Both legs, arms, and trunk	4 months	Left side of the body
Keipert and Nurse (1971) [15]	NS	M	Birth	Legs, right arm, and right trunk	2 months	Right arm and right calf
South and Jacobs (1978) [16]	NS	F	Birth	Legs, feet, back, and chest	NS	Right side of the body
Spraker et al. (1984) [17]	Caucasian	F	2 weeks	Left side of the face and trunk	2 weeks	Entire left side of the body
Avci et al. (2001) [18]	Turkish	F	Birth	Most part of the body	NS	Right lower limb
Levy and Lam (2011) [19]	NS	F	Birth	Right thigh and right shin	3 months	Right thigh
de Maio et al. (2014) [20]	Italian	F	Birth	Whole body	NS	Left lower limb
Amaral et al. (2018) [21]	NS	M	Birth	Right side of the body, left hand, and left malleolus	6 months	Right lower limb
*Present cases*						
Case 1	Indian	F	Birth	Left lower limb	Birth	Left lower limb
Case 2	Chinese	F	Birth	Right lower limb	Birth	Right lower limb

Abbreviations: CMTC = cutis marmorata telangiectatica congenita; NS = not specified; M = male; F = female.
